# Comparisons of between-group differentiation in male kinship between bonobos and chimpanzees

**DOI:** 10.1038/s41598-019-57133-z

**Published:** 2020-01-14

**Authors:** Shintaro Ishizuka, Hiroyuki Takemoto, Tetsuya Sakamaki, Nahoko Tokuyama, Kazuya Toda, Chie Hashimoto, Takeshi Furuichi

**Affiliations:** 1 Primate Research Institute, Kyoto University, Inuyama, Japan; 2 Japan Society for the Promotion of Science, Chiyoda-ku, Japan; 3 Antwerp Zoo Foundation, Antwerp, Belgium; 4 Department of Evolutionary Studies of Biosystems, The Graduate University for Advanced Studies, Yokosuka, Japan

**Keywords:** Ecology, Evolution, Genetics, Molecular biology, Zoology

## Abstract

Patterns of kinship among individuals in different groups have been rarely examined in animals. Two closest living relatives of humans, bonobos and chimpanzees share many characteristics of social systems including male philopatry, whereas one major difference between the two species is the nature of intergroup relationship. Intergroup relationship is basically antagonistic and males sometimes kill individuals of other groups in chimpanzees, whereas it is much more moderate in bonobos and copulations between individuals of different groups are often observed during intergroup encounters. Such behavioural differences may facilitate more frequent between-group male gene flow and greater between-group differentiation in male kinship in bonobos than in chimpanzees. Here we compared differences between average relatedness among males within groups and that among males of neighbouring groups, and between-group male genetic distance between bonobos and chimpanzees. Contrary to expectation, the differences between average relatedness among males within groups and that among males of neighbouring groups were significantly greater in bonobos than in chimpanzees. There were no significant differences in autosomal and Y-chromosomal between-group male genetic distance between the two species. Our results showed that intergroup male kinship is similarly or more differentiated in bonobos than in chimpanzees.

## Introduction

Kinship is one of key factors to affect affinity among animals^1^. It has been clarified that reciprocity and cooperation in animals have been attributed to kin selection, a process whereby individuals cooperate with relatives and gain indirect fitness benefits through the reproduction of kin^2^. Researchers have clarified that kin selection can occur within social groups of each species. However, patterns of kinship among individuals in different groups have been rarely examined in especially large mammalian species (e.g. chimpanzee^3^, western lowland gorilla^4^), since it is tough to examine them in the wild. For a better understanding of the influences of kinship on social interactions in animals, patterns of kinship among individuals in different groups should be investigated with references about patterns of social interactions among them.

Comparisons between bonobos and chimpanzees are effective in examining the relationships between intergroup interactions and kinship among individuals in different groups. These two species share many characteristics of their social system including male philopatry, multi-male/multi-female group composition, and fission–fusion dynamics^5–7^. Despite these similarities, the nature of intergroup relationship largely differs between the two species. Intergroup relationship in chimpanzees is basically antagonistic and male chimpanzees sometimes make lethal coalitionary attacks and kill males of different groups^8–11^. Contrary to chimpanzees, bonobos show much more moderate intergroup relationships. Although intergroup male-male relationships are antagonistic, male bonobos rarely show lethal attacks towards those of different groups^12–14^. Furthermore, non-antagonistic intergroup encounters, in which members of different groups occasionally mingle peacefully, are often led by females^15^. Even copulations between individuals of different groups are often observed during the encounters^12^. Thus, breeding between individuals of neighbouring groups may be more frequent in bonobos than in chimpanzees. This is also suggested by several genetic studies, as evidence of extra-group paternity was found in only one of four sites for chimpanzees^16–21^, whereas potential cases of extra-group paternity were found in all three study sites for bonobos^22–24^. In addition, the number of available cases for male migration between groups is larger in bonobos than in chimpanzees^25–27^. These differences between the two species suggest that between-group male gene flow may be more frequent in bonobos than in chimpanzees, which is also implied by a previous study using mathematical simulations^28^.

Compared to chimpanzees, bonobos potentially have more frequent between-group male gene flow, which may enable smaller between-group differentiation in male kinship in bonobos than in chimpanzees. A few previous studies partially showed that relatedness among males within groups tends to be higher than that among males of neighbouring groups in both species^24,29^. However, data are still scarce for both species and problematically, a direct comparison for relatedness among males within groups or of neighbouring groups, and between-group male genetic distance has not been conducted between the two species. To clarify whether differences in intergroup interactions between the two species may be reflected in patterns of intergroup male kinship, the extent of between-group differentiation in male kinship should be directly compared between the two species using empirical data.

In this study, we analysed autosomal microsatellite genotypes and estimated pairwise relatedness for male chimpanzees of two neighbouring groups in the Kalinzu Central Forest Reserve. We also estimated pairwise relatedness for male chimpanzees of the three neighbouring groups in the Tai forest, and male bonobos of three neighbouring groups at Wamba, referring to autosomal microsatellite genotype data from previous studies^24,29^. Using data for the five and three neighbouring groups of chimpanzees and bonobos, we compared differences between average relatedness among males within groups and that among males of neighbouring groups. Secondly, we determined Y-chromosomal haplotypes for male chimpanzees of the two neighbouring groups in Kalinzu, and male bonobos of the three neighbouring groups at Wamba by analysing Y-chromosomal microsatellite loci. Using data for autosomal genotypes and Y-chromosomal haplotypes, we calculated the male fixation index, as the measure of between-group male genetic distance, among the two neighbouring groups of chimpanzees and the three neighbouring groups of bonobos. Adding data for male fixation index from a previous study^28^, we compared the extent of between-group male genetic distance between the two species.

## Results

### Relatedness among males in groups or of neighbouring groups

**Figure 1:**
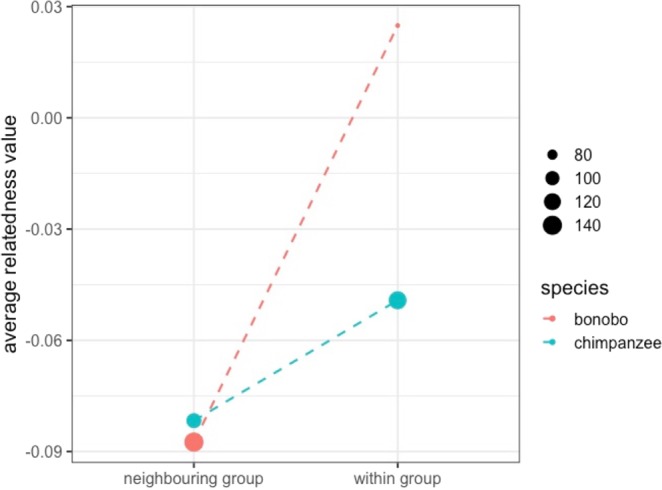
Average relatedness values among males within groups and that among males of neighbouring groups. “Within group” and “neighbouring group” represent the average relatedness values among males within groups and that among males of neighbouring groups, respectively. Circle sizes represent the number of dyads.

Average relatedness among males within three out of five chimpanzee groups, and three all bonobo groups showed higher values than that among males of neighbouring groups (Supporting Information, Fig. S1). Average relatedness among male bonobos in the E1 and PW groups was significantly higher than that among males of neighbouring groups (permutation test, *p* < 0.01). Average relatedness among male bonobos within all the groups was significantly higher than that among males of the neighbouring groups (permutation test, *p* < 0.01; Fig. 1). On the other hand, average relatedness among male chimpanzees within all the groups showed a higher value than that among males of the neighbouring groups but the difference was not significant (permutation test, *p* = 0.21; Fig. 1). The relationship between the dyad category (within group/neighbouring group) and average relatedness value was significant between the two species (power for the predictor = 22.3%, 19.8–25.0% with 95% confidence interval, Table 1).

**Table 1:** Results of the GLMM.

	Estimate	Std. Error	df	t value	Pr (>|t|)
(Intercept)	0.031	0.033	5.1	0.93	0.40
Species	−0.087	0.032	7.2	−2.7	<0.05
dyad category	−0.12	0.021	854.1	−5.5	<0.001
number of males in groups	2.75e-04	0.0031	3.1	0.087	0.94
species × dyad category	0.082	0.030	728.4	2.75	<0.01

Species, dyad category, and number of males in groups represent the difference between species, whether the dyad is between males within groups or those of neighbouring groups, number of males in groups. Species × dyad category represent two-way interaction between species difference and dyad category.

### Between-group male genetic distance

**Figure 2:**
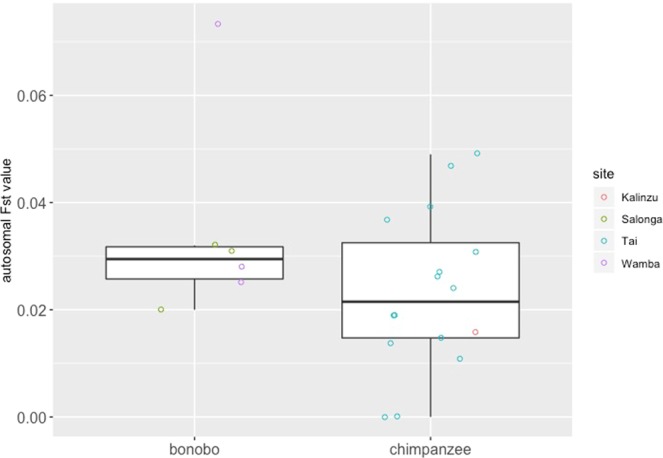
Autosomal Fst values between neighbouring groups of bonobos and chimpanzees.

**Figure 3:**
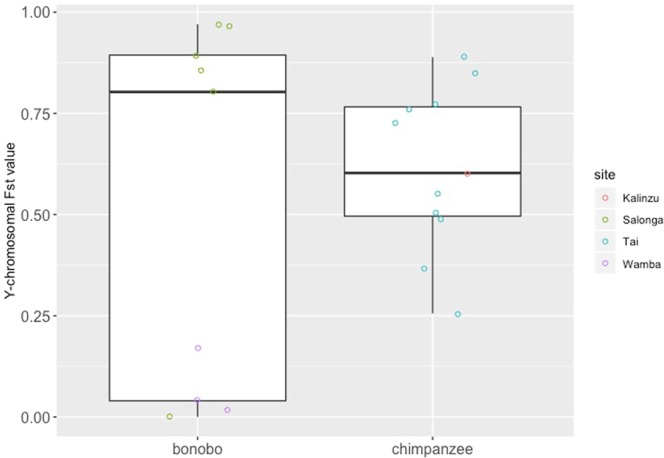
Y-chromosomal Fst values between neighbouring groups of bonobos and chimpanzees.

Five Y-chromosomal haplotypes were found in male chimpanzees of the two neighbouring groups (M and S) in Kalinzu (Supporting Information, Table S1), and four Y-chromosomal haplotypes were found in male bonobos of the three neighbouring groups (E1, PE and PW) at Wamba (Supporting Information, Table S2). Mean values of autosomal between-group male genetic distance in bonobos and chimpanzees were 0.035 ± 0.019 and 0.023 ± 0.015, respectively. The difference between the two species was not significant (*U* test, *p* = 0.30, power = 0.31; Fig. 2, Supporting Information, Table S3). Mean values of Y-chromosomal between-group male genetic distance in bonobos and chimpanzees were 0.52 ± 0.45 and 0.62 ± 0.20, respectively. The difference between the two species was not significant (*U* test, *p* = 0.58, power = 0.11; Fig. 3, Supporting Information, Table S4).

## Discussion

Average relatedness among males in three out of five chimpanzee groups and all bonobo groups showed higher values than that among males of neighbouring groups (Supporting Information, Fig. S1). These results were not inconsistent with the expectation that it originates from male philopatry; specifically, males within groups are expected to be more closely related with each other than males belonging to different groups. On the other hand, when all groups of each species are pooled, only bonobos showed the statistical significant difference between average relatedness among males within groups and that among males of neighbouring groups. The absence of the significant difference in chimpanzees might be due to idiosyncratic reasons for the two chimpanzee groups. In the North community of chimpanzees, which is one of the two groups, there were only two males in the community, and they might not be related to each other. The North community experienced a drastic decrease in community size from 1982 to 1996^30^, which might have left only two unrelated males in the community. In the M group of chimpanzees, which is another of the two groups, there were 15 alive adult males in the group. A previous study also showed that high average relatedness among philopatric males can be expected only when the number of males is small in groups^29^. Because the M group consisted of 15 adult males, which is relatively numerous compared to other chimpanzee groups^5,30–33^, average relatedness among males in the M group might be low and similar to that among males of neighbouring groups. These may contribute to the absence of significant difference between average relatedness among males within groups and that among males of the neighbouring groups in chimpanzees.

The species difference in the relationship between the dyad category and average relatedness value was significant (Table 1). Previous studies indicated that male reproductive skew was higher in bonobos than in chimpanzees^23,24^, which was mainly because reproductive success of males was largely affected by their mothers in bonobos but not in chimpanzees^34^. Higher male reproductive skew in bonobos is expected to increase relatedness among males within bonobo groups rather than within chimpanzee groups. In comparison, between-group male gene flow is rare in both species, even though it is potentially more frequent in bonobos. When between-group male gene flow is rare in male-philopatric species, average relatedness among males of different groups is expected to be low. In fact, average relatedness among males of neighbouring groups showed lower values than those among females within groups or of neighbouring groups in both bonobos and chimpanzees^24,29^. Thus, males within groups are more closely related in bonobos than in chimpanzees, whereas those of neighbouring groups are rarely related in either species. Consequently, the differences between average relatedness among males within groups and that among males of neighbouring groups were considered to be significantly greater in bonobos than in chimpanzees. In addition, although a recent study showed that female bonobos often migrate into neighbouring groups^35^, the effects of such female migration might not be enough strong to increase average relatedness among males of neighbouring groups.

Significant differences were not observed in autosomal and Y-chromosomal between-group male genetic distance between the two species; however, the power of the statistical tests was not strong (Figs. 2, 3). Given the absence of significant differences in between-group male genetic distance, it remains unclear whether between-group differentiation in male kinship was greater in bonobos or in chimpanzees. However, the results showing that bonobos had a higher mean value of autosomal between-group male genetic distance than chimpanzees (bonobo: 0.035 ± 0.019, chimpanzee: 0.023 ± 0.015) were not inconsistent with greater differences between average relatedness among males within groups and that among males of neighbouring groups in bonobos. In comparison, values of Y-chromosomal male genetic distance between bonobo groups at Wamba were extremely low (under 0.2, Fig. 3). Although the reason for this phenomenon remains unclear, the results might have been influenced by the low genetic diversity in male bonobos at Wamba. In our analysis, only four Y-chromosomal haplotypes were found among the 21 male bonobos in the three groups. Because Y-chromosomal haplotype diversity is low in the Wamba population, male bonobos from the three groups shared Y-chromosomal haplotypes. Consequently, Y-chromosomal between-group male genetic distance might below.

Our results are inconsistent with the expectation come from less intensive intergroup male aggressions in bonobos compared to chimpanzees. Previous studies proposed evidence that patterns of alliance formation or cooperative interactions among males were not explained by kinship in groups of male-philopatric species (e.g. chimpanzee^36^, bottlenose dolphin^37^). In male-philopatric species, kinship may basically unexplain patterns of social interactions among males of both same and different groups. One alternative explanation for the difference in antagonism towards males of different groups between the two species is that prolonged period of oestrus in female bonobos may reduce intensity of intergroup aggression in male bonobos^38^. Female bonobos show longer periods of sexual receptivity during their life span compared to female chimpanzees, which leads to a larger number of simultaneously receptive females in bonobo groups. Contrary to male chimpanzees, which often demonstrate intergroup aggression to obtain females from neighbouring groups^39,40^, male bonobos may not need to recruit females from other groups because of the large number of receptive females in their own group. Another possible explanation for a more moderate intergroup aggression in male bonobos is the fact that bonobos can forage with males of different groups. When bonobo groups encounter, they often merge and forage together^12,13^, which may eliminate the necessity to defend their territory. Moreover, bonobos rely more on terrestrial herbaceous vegetation for their diet than chimpanzees^41,42^. This suggests that fruits may be less important in the diet of bonobos than in the diet of chimpanzees. This may reduce the necessity to defend their territory. The presence of lethal intergroup aggression in male chimpanzees might also be explained by the large variations in party size and composition, which cause an imbalance in the fighting abilities of a group during intergroup encounters^39,40^. A previous study in which parties were followed for one hour reported that almost half of the group members were found in bonobo parties, while a smaller proportion of members were found in chimpanzee parties^43^. This suggests that there is larger variation in the daily party size in chimpanzees compared to bonobos. Variation in party size can lead to encounters between groups of significant size difference. During such encounters, male chimpanzees from the larger party may launch lethal coalitionary attacks, and kill males of the other groups.

We clarified fine-scale male genetic structures in bonobos and chimpanzees. Our data is valuable in that fine-scale genetic structures for large mammals have been rarely investigated. Furthermore, our results showed that intergroup male kinship is similarly or more differentiated in bonobos than in chimpanzees. Future studies are required to obtain empirical data on the links between intergroup interactions and patterns of intergroup male kinship in the two species.

## Methods

### Study subjects

One study subjects were male chimpanzees of two neighbouring groups (M and S) in the Kalinzu Forest Reserve, Republic of Uganda^44,45^. All adult males of the two groups have been identified since 2014. At the time of the present study, the M and S group consisted of approximately 100 individuals including 15 adult males, and approximately 30 individuals including 5 adult males, respectively. Age classification for each individual was followed by previous studies^30,46^.

Another study subjects were male bonobos of three neighbouring groups (E1, PE and PW) at Wamba in the Luo Scientific Reserve area, Democratic Republic of the Congo^47,48^. All individuals of the three groups have been identified since 2014. At the time of the present study, the E1, PE, and PW group consisted of 37 individuals including 10 adult or adolescent males, 26 individuals including 6 adult or adolescent males, and 14 individuals including 5 adult or adolescent males, respectively. Age classification for each individual was followed by a previous study^49^.

### DNA sampling, extraction, and semi-quantification

Faeces of chimpanzees for genetic analysis were collected using cotton swabs and conserved in lysis buffer at ambient temperature^50^. A total of 47 samples were used for analysis. Samples of bonobos were collected in our previous works^24,35,51^. We performed DNA extractions on the samples of which the identity of the individuals had been confirmed.

DNA was extracted using a QIAamp Stool Mini Kit for most samples (Qiagen, CA, USA). For some samples, DNA was extracted during a previous study using a Wizard SV Gel and PCR Clean-Up System (Promega, Madison, IW, USA). The applicability of the obtained DNA extraction was tested using a verification system designed in a previous study^50^. DNA from the obtained samples was amplified semi-quantitatively targeting the c-*myc* proto-oncogene^52^. DNA from human placenta (SIGMA) with known concentration was used as a positive control. By comparing the intensity of PCR bands after electrophoresis we categorised the quantity of the samples as follows: >500 pg/μl, >300 pg/μl, >100 pg/μl, and ≦ 100 pg/μl.

### Genetic analysis

The genotypes at eight autosomal microsatellite loci for the male chimpanzees in Kalinzu were newly analysed^24,53,54^. The genotypes at autosomal microsatellite loci for male chimpanzees in the Tai forest and the male bonobos at Wamba were referred from previous studies^24,29^. 12 Y-chromosomal loci for the male chimpanzees in Kalinzu and male bonobos at Wamba were also analysed^55–57^. Because DNA of bonobos was not successfully amplified for two of the 12 loci, their Y-chromosomal haplotypes were determined with other 10 loci.

To analyse genotypes for the male chimpanzees in Kalinzu at eight autosomal microsatellite loci, two primer sets: set A-1 (D11s2002, D7s817, D9s910, and D6s493) and set A-2 (D2s1326, D14s306, D5s1457, and D12s66) were prepared. Another two primer sets: Y-1 (DyS439, DyS469, DyS520, DyS533, DyS562, and DyS632), and set Y-2 (DyS392, DyS510, DyS517, DyS588, DyS612, and DyS630) were prepared for the Y-chromosomal genetic analysis.

Genotyping was carried out using two-step multiplex PCR^54^. In the first step, multiplex PCRs were performed in 13-μl reaction volumes comprising 1 μl of extracted faecal DNA, 200 nM of each forward and reverse non-labelled primer (each set), 2× buffer, 0.4 mM of each dNTP, and 0.5 U of KOD FX polymerase (Toyobo, Tokyo, Japan). Amplification parameters were as follows: 94 °C for 5 min; 35 cycles of 94 °C for 30 s, 64 °C for 30 s, and 68 °C for 1 min; and a final 5-min extension at 66 °C. In the second step, we carried out singleplex PCR amplifications. Singleplex PCRs were performed in 13-μl reaction volumes comprising 1 μl of multiplex PCR products, 50 to 100 nM of forward primer, fluorescently labelled by FAM or HEX, and of reverse primer, 2× buffer, 0.4 mM of each dNTP, and 0.5 U of KOD FX polymerase. Amplification parameters were as follows: 94 °C for 5 min; 45 cycles of 94 °C for 30 s, 64 °C for 30 s, and 68 °C for 1 min; and a final 5-min extension at 66 °C. The amplification products were separated using capillary electrophoresis, using an ABI 3130xl Genetic Analyzer (Applied Biosystems, CA). Alleles were sized using GeneMapper Software v4.0 (Applied Biosystems).

To obtain accurate genotypes at microsatellite loci, we repeated genotyping for several times following the recommendations of previous studies^52,54^. We identified homozygotes when the single allele was repeatedly observed at least three times in samples whose concentrations were more than 100 pg/μl, and at least four times in samples whose concentrations were less than 100 pg/μl. We identified heterozygotes when each allele was repeatedly observed more than twice at each locus.

### Analysis of relatedness among males

We estimated pairwise relatedness values among all adult male chimpanzees in Kalinzu (15 in the M, and 5 in the S). We also estimated pairwise relatedness values among all adult and adolescent male chimpanzees of the three neighbouring communities in Tai forest (2 in the North, 3 in the Middle, and 5 in the South), since adolescent male chimpanzees in Tai forest show behaviours that are typically observed in adult males^30,58,59^, and were included in the analysis of adult males in a previous study^29^. Ages of individuals were classified at 2001, because the number of resident males differed between communities at the time. We also estimated pairwise relatedness values among adult and adolescent male bonobos at Wamba (10 in the E1, 6 in the PE, and 5 in the PW), since adolescent male bonobos are also engaged in aggressive interactions with other adult males and involved in male dominance hierarchy^24,60^. The pairwise relatedness values for all the possible pairs among males within groups or of neighbouring groups were estimated using the Queller and Goodnight estimator implemented in GeneAlEx v6.3^61^. Although this method often causes errors in estimation for exact dyadic relatedness values^62,63^, average relatedness values among individuals of different categories can be compared with reasonable accuracy^61,62^. To compare the average relatedness value between categories, a permutation test was conducted in R v3.3.3. A p value was obtained by 999 permutations.

We ran a Generalized Liner Mixed Model with a normal error structure to determine the effects of species difference on the differences between average relatedness among males within groups and that among males of neighbouring groups. The model was fitted in R v3.6.1 using the R-package “lme4”^64^. For each male, we determined the following information: species (bonobo/chimpanzee), age class (old: >35 years, middle: 20–35 years, young: <20 years), group ID, number of males in the group, pairwise relatedness value with other males, and category of the male-male dyad (within group/neighbouring group). As the test predictor, we included the two-way interaction between species and the category of the male-male dyad. We also included the number of males in groups, and the age class of males as fixed effects to control for their effects on male relatedness. This is because the number of males within groups and ages of males have been shown to potentially affect patterns of pairwise relatedness among males within groups in male-philopatric species^20,29^. We also included male ID and group ID as random effects. We constructed six candidate models and null model, and calculated the Akaike’s information criterion (AIC) for each model (Supporting Information, Table S5). Comparing the AIC values, we selected the best fitted model. The best fitted model included the two-way interaction between species and the category of the male-male dyad, as well as the number of males in groups as predictor variables. Statistical power for the predictors was calculated from 999 simulations using the R-package “simr”^65^.

### Analysis of between-group male genetic distance

We used the Arlequin v3.5 and calculated the autosomal and Y-chromosomal fixation indices, as a measure of genetic distance between groups, based on autosomal genotypes and Y-chromosomal haplotypes for the male chimpanzees in Kalinzu, and male bonobos at Wamba. To increase the dataset, we referred to a previous study^28^, in which both the autosomal and Y-chromosomal male fixation indices among neighbouring groups were investigated in both species, although not all males of each group might have been sampled. The data of each group included at least four samples. We added these data to create a larger-scaled comparison between the two species for analysis. We conducted the two-sided Wilcoxon-Mann-Whitney test and calculated the power for the test in R v3.3.3 using the R-package “wmwpow”.

### Ethical approval

This study was approved by the Uganda National Council for Science and Technology, the Uganda Wildlife Authority, the National Forestry Authority of Uganda, the Research Centre for Ecology and Forestry, and the Ministry of Scientific Research of the Democratic Republic of the Congo. All laws in Republic of Uganda and Democratic Republic of the Congo were followed. This study was conformed to the Guidelines for Field Research established by the Ethics Committee of the Primate Research Institute, Kyoto University.

## Supplementary information

Supplementary information.
