# Supplementary material for: Comparisons of between-group differentiation in male kinship between bonobos and chimpanzees
Source: Sci Rep. 2020 Jan 14;10:251. doi: 10.1038/s41598-019-57133-z (PMC6959343; doi:10.1038/s41598-019-57133-z)
Supplement: Supplementary file 1 — Supplementary information. [file 41598_2019_57133_MOESM1_ESM.pdf]

Supporting Information

Comparisons of between-group differentiation in male kinship between bonobos and chimpanzees

Shintaro Ishizuka[1,2]*, Hiroyuki Takemoto[1], Tetsuya Sakamaki[1,3], Nahoko Tokuyama[1,2,4], Kazuya Toda[1,2], Chie Hashimoto[1] & Takeshi Furuichi[1]

1.Primate Research Institute, Kyoto University
2.Japan Society for the Promotion of Science
3.Antwerp Zoo Foundation
4.Department of Evolutionary Studies of Biosystems, The Graduate University for Advanced Studies

Figure S1: Average relatedness values among males within each group and that among males of neighbouring groups in bonobos and chimpanzees. “within group” and “neighbouring group” represent average relatedness value among males within groups and that among males of neighbouring groups, respectively. Circle sizes represent the number of dyads.

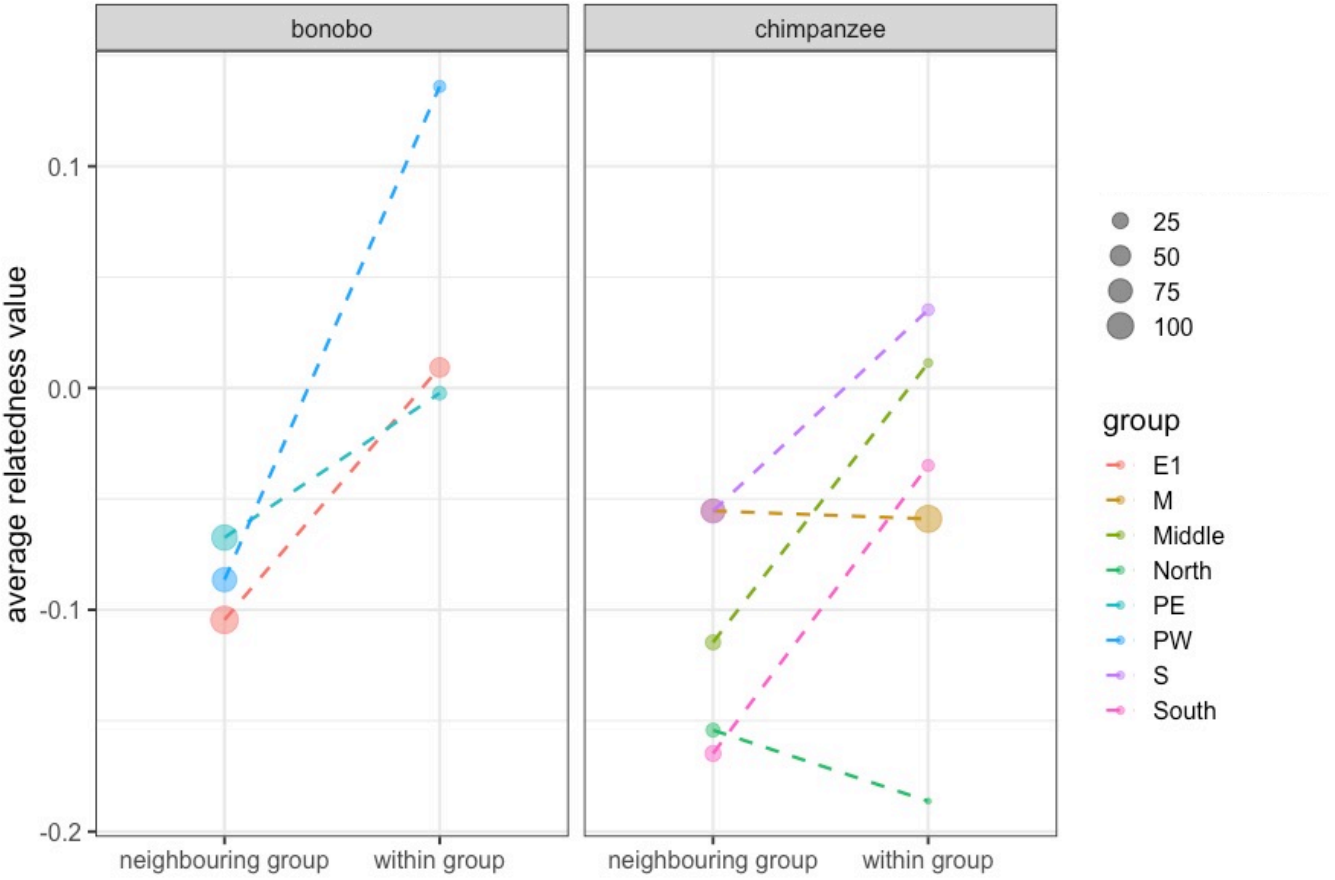

Table S1: Y-chromosomal haplotypes in chimpanzees in Kalinzu Central Forest Reserve. “HP ID” indicates the name of the haplotype. Allele types at each locus are given as arabic numbers. “Number” indicates the number of individuals sharing the haplotype in each group (M and S).

HP ID	DyS 439	DyS 469	DyS 533	DyS 562	DyS 632	DyS 520	DyS 588	DyS 510	DyS 517	DyS 612	DyS 630	DyS 392	Number	
													M	S
A	1	1	1	1	1	1	1	1	1	1	1	1	13	0
B	1	1	1	1	1	2	1	1	1	1	1	1	1	0
C	1	1	1	1	1	1	1	2	1	1	2	1	1	1
D	1	1	1	1	1	1	1	2	1	1	3	1	0	3
E	1	2	2	1	1	1	1	1	2	2	2	1	0	1

Table S2: Y-chromosomal haplotypes in bonobos at Wamba. “HP ID” indicates the name of the haplotype. Allele types at each locus are given as arabic numbers. “Number” indicates the number of individuals sharing the haplotype in each group (E1, PE, and PW).

HP ID	DyS 439	DyS 469	DyS 533	DyS 562	DyS 632	DyS 520	DyS 510	DyS 517	DyS 612	DyS 392	Number		
											E1	PE	PW
A	1	1	1	1	1	1	1	1	1	1	6	6	4
B	2	1	1	1	1	1	1	2	1	1	3	0	0
C	2	1	1	2	1	1	1	2	1	1	1	0	0
D	1	1	1	1	2	1	1	1	1	1	0	0	1

Table S3: List of autosomal fixation index between neighbouring groups of the two species. Sample size in each group is indicated within brackets.

Species	Fst value	Pair of groups	Reference
Bonobo	0.028	E1 (10) & PE (6)	This study
	0.074	E1 (10) & PW (5)	This study
	0.025	PE (6) & PW (5)	This study
	0.032	C1 (11) & C2 (10)	Schubert *et al*. 2011
	0.02	C2 (10) & C4 (9)	Schubert *et al*. 2011
	0.031	C4 (9) & C1 (10)	Schubert *et al*. 2011
Chimpanzee	0.016	M (15) & S (5)	This study
	0.031	East (10) & GTZ (8)	Schubert *et al*. 2011
	0.015	East (10) & Meteo (6)	Schubert *et al*. 2011
	0.047	East (10) & Middle (4)	Schubert *et al*. 2011
	0.011	East (10) & North (9)	Schubert *et al*. 2011
	0.019	East (10) & South (26)	Schubert *et al*. 2011
	0	GTZ (8) & Meteo (6)	Schubert *et al*. 2011
	0.039	GTZ (8) & Middle (4)	Schubert *et al*. 2011
	0.027	GTZ (8) & North (9)	Schubert *et al*. 2011
	0.019	GTZ (8) & South (26)	Schubert *et al*. 2011
	0.049	Meteo (6) & Middle (4)	Schubert *et al*. 2011
	0.014	Meteo (6) & North (9)	Schubert *et al*. 2011
	0	Meteo (6) & South (26)	Schubert *et al*. 2011
	0.026	Middle (4) & North (9)	Schubert *et al*. 2011
	0.037	Middle (4) & South (26)	Schubert *et al*. 2011
	0.024	North (9) & South (26)	Schubert *et al*. 2011

Table S4: List of Y-chromosomal fixation index between neighbouring groups of the two species. Sample size in each group is indicated within brackets.

Species	Fst value	Pair of groups	Reference
Bonobo	0.169	E1 (10) & PE (6)	This study
	0.017	E1 (10) & PW (5)	This study
	0.04	PE (6) & PW (5)	This study
	0.97	C2 (15) & C1 (10)	Schubert *et al*. 2011
	0.964	C2 (15) & C4 (6)	Schubert *et al*. 2011
	0.803	C2 (15) & C3 (6)	Schubert *et al*. 2011
	0	C1 (10) & C4 (6)	Schubert *et al*. 2011
	0.894	C1 (10) & C3 (6)	Schubert *et al*. 2011
	0.856	C4 (6) & C3 (6)	Schubert *et al*. 2011
Chimpanzee	0.603	M (15) & S (5)	This study
	0.368	East (8) & GTZ (6)	Schubert *et al*. 2011
	0.256	East (8) & Middle (4)	Schubert *et al*. 2011
	0.488	East (8) & North (4)	Schubert *et al*. 2011
	0.504	East (8) & South (15)	Schubert *et al*. 2011
	0.758	GTZ (6) & Middle (4)	Schubert *et al*. 2011
	0.851	GTZ (6) & North (4)	Schubert *et al*. 2011
	0.551	GTZ (6) & South (15)	Schubert *et al*. 2011
	0.889	Middle (4) & North (4)	Schubert *et al*. 2011
	0.725	Middle (4) & South (15)	Schubert *et al*. 2011
	0.774	North (4) & South (15)	Schubert *et al*. 2011

Table S5: Comparisons of GLMMs using different predictor variables. Circles indicate that the predictor variable was used in the model.

	Predictor variable					AIC
	Species	Dyad category	Species × dyad category	Age class	Number of males in the group	
Null model						−279.6
Model 1	○	○		○	○	−298.0
Model 2	○	○		○		−298.5
Model 3	○	○			○	−299.3
Model 4	○	○	○	○	○	−302.7
Model 5	○	○	○	○		−296.9
Model 6	○	○	○		○	−304.1
